# Under-the-Flap Crosslinking and LASIK in Early Ectasia with Hyperopic Refractive Error

**DOI:** 10.1155/2018/4342984

**Published:** 2018-11-15

**Authors:** Sylvain el-Khoury, Youssef Abdelmassih, Mazen Amro, Elias Chelala, Elias Jarade

**Affiliations:** ^1^Beirut Eye Specialist Hospital, P.O. Box 116-5311, Al-Mathaf Square, Beirut, Lebanon; ^2^Saint-Joseph University, Faculty of Medicine, Beirut, Lebanon; ^3^School of Medical Sciences, Lebanese University, Beirut, Lebanon; ^4^Mediclinic Dubai Mall, Dubai, UAE

## Abstract

**Purpose:**

To present safety, efficacy, and early results of a new combinational treatment for early corneal ectasia with hyperopic refractive error aimed to reinstate emmetropia and stabilize cornea.

**Method:**

This is a retrospective case series. All surgeries were performed at the Beirut Eye Specialist Hospital, Lebanon. Surgical procedure consisted of (1) lifting flap (post-LASIK ectasia)/creation of corneal flap (keratoconus), (2) application of excimer laser ablation to correct refractive error, (3) loose repositioning of flap, (4) under-the-flap irrigation with riboflavin 0.1% dextran solution, and (5) application of UVA light.

**Results:**

A total of 7 eyes (4 patients; mean age 24.25 years; all male) were included. 2 patients had early keratoconus, and 2 patients had early post-LASIK ectasia. Pretreatment vs. last postoperative follow-up visit (mean 11.25 months; range 6–15 months) UDVA (logMAR), spherical equivalent (SE) (D), astigmatism (D), and central pachymetry (*µ*m) were 0.35 ± 0.18 vs. 0.05 ± 0.07, *p*=0.017; −0.81 ± 0.67 vs. −0.46 ± 0.57, *p*=0.078; 2.46 ± 0.53 vs. 0.68 ± 0.28, *p*=0.018; and 547 ± 58 vs. 536 ± 49, *p*=0.07, respectively. In all eyes, BCVA was 0.1 logMAR or better before and after treatment. No eye showed a decrease in BCVA. Two eyes of one patient had an epithelial ingrowth, which was removed in one case. Follow-up results showed no major complications and no progression of corneal ectasia.

**Conclusion:**

Early results showed that under-the-flap CXL with excimer laser correction is an effective treatment for early hyperopic keratectasia, with the advantage of rapid recovery, postoperative corneal stability, and no epithelial healing complications. The procedure seems to bear a risk for postoperative epithelial growth into the flap interface.

## 1. Introduction

Keratoconus and post-LASIK ectasia are progressive, noninflammatory corneal diseases that lead to an outward bulging (cone) and to a thinning of the cornea. Both conditions behave similarly and are due to a biomechanically altered cornea that cannot withhold intraocular pressure and hence a cone-shaped outward bulging develops. Post-LASIK ectasia is an iatrogenic condition, where changes of the biomechanical properties are due to a surgical weakening of the cornea, whereas keratoconus is a multifactorial disease with environmental and genetic risk factors [1–3].

Both conditions have a similar clinical presentation: the cone usually leads to a progressive myopic shift in spherical equivalent (SE). In some cases, however, the cone may also lead to a hyperopic shift in SE [4]. It has been shown that the direction of the refractive shift is mainly dependent on the cone location and on the severity of the disease, with a hyperopic SE observed in cases where the cone is located very far from the visual axis and in mild to moderate cone volumes [5].

When it comes to the treatment of hyperopic ectasia, the traditional approach has been the stabilization of the cornea via crosslinking and a secondary external refraction correction (spectacles/contact lenses). But this therapy option has its limitations and may lead, e.g., in the case of severe residual anisometropia, to unsatisfactory outcomes. Furthermore, the expectations of patients who wish to remain spectacle-free cannot be met.

Looking for alternative treatment options that reinstate emmetropia, combined photorefractive keratectomy and crosslinking (PRK-CXL) has to be evaluated, since it has been described to be safe and effective in treating myopic refractive error in early keratoconus [6–8]. However, PRK is not very desirable in hyperopic ablation as it has a risk of peripheral corneal scarring, haze, and hyperopic regression [9, 10]. On the other hand, LASIK has been shown to lead to satisfactory results in hyperopia and hyperopic or mixed astigmatism [11], and when compared to photorefractive keratectomy (PRK) for hyperopia, LASIK was also associated with a faster stabilization of refraction, less pain [12], and less hyperopic regression, but comparable efficacy [13].

To prevent the development of post-LASIK ectasia in eyes at high risk, Kanellopoulos et al. introduced the LASIK Xtra technique (Avedro, Massachusetts, USA) in 2012 [14]. In this technique, prophylactic high irradiation CXL is performed in adjunction to the LASIK procedure and without deepithelisation. Several studies demonstrated this procedure to be safe and effective, to show less regression and to have less discomfort [15–17].

In this retrospective case series, we present the outcomes of a new method for the treatment of early ectasia with hyperopic SE, performed in seven eyes of four patients with a maximum follow-up of 15 months. The new method is a combinational procedure of an under-the-flap excimer laser ablation followed by an under-the-flap CXL in the same session. It stems from the satisfactory results obtained by LASIK for the correction of hyperopia and from the imperative of CXL in early and progressive corneal ectasia, irrespective of whether the ectasia is due to keratoconus or to LASIK surgery. For this treatment, there is no need to produce a new flap in post-LASIK ectasia, and the old flap can simply be relifted, but in keratoconus, a new flap has to be created using a femtosecond laser.

## 2. Methods

All cases included in this study had early corneal ectasia with a major hyperopic component of refractive error (hyperopia, hyperopic astigmatism, and mixed astigmatism), a good corrected distance visual acuity (CDVA), and a clear cornea in a relatively good condition. No other ocular pathologies were present. Diagnosis of keratoconus was based on a combination of computed slit-scanning videokeratography of the anterior and posterior corneal surface, keratometric readings, and corneal pachymetry (Wavelight Allegro Oculyzer, Alcon Laboratories, Inc., Fort Worth, TX) [18, 19]. Keratoconus stage was classified according to the Amsler–Krumeich criteria [20]. Indication for surgery was stabilization of the cornea and the aim to achieve emmetropia. For both keratoconus patients, emmetropia with good UDVA was needed for occupational purposes. Post-LASIK ectasia patients were unhappy with their eyeglasses and were aiming for a spectacle-free lifestyle. Patients were treated between December 2014 and September 2015 at the Beirut Eye Specialist Hospital, Lebanon.

Treatment consisted of flap lifting/creation, excimer stromal laser ablation followed by repositioning of the flap, irrigation of the interface with riboflavin, and UV exposure. All surgeries were performed by the same surgeon (E.J.) under topical anesthesia. All patients had to sign an informed consent for surgery. The study was approved by the institutional review board. In the case of post-LASIK ectasia, the old corneal flap was lifted again. In the case of keratoconus, a new corneal flap was produced at a depth of 90 *µ*m using a femtosecond laser (IntraLase FS60, Abbot, Abbot Park, Illinois, USA). After lifting the flap, the excimer laser (Allegretto Wave, Alcon, Fort Worth, USA) was applied to the midperipheral and, where necessary, to the central corneal stroma. Calculations were made in order not to exceed 20 *μ*m of central excimer laser ablation. After laser ablation, the flap was loosely repositioned and a mixed solution of riboflavin 0.1% dextran was used to irrigate the interface and kept for 10 minutes. The interface was then quickly irrigated with saline solution, the flap was properly readjusted, and fluid was removed using Weck-Cel® (Beaver-Visitec International, USA), a dry cellulose sponge. Subsequently, UV light (radiant energy of 3.0 ± 0.3 mW/cm^2^; UV-X illumination system, version 1000; IROC AG, Zurich, Switzerland) was focused on the corneal apex from a distance of 5 cm for 30 minutes, while dropping riboflavin 0.1% dextran solution every 2 minutes. Postoperatively, patients received acetaminophen 500 mg twice daily for 3 days and 1 drop of gatifloxacin 0.3% 6 times daily for 7 days with 1 drop of tobramycin 0.3%-dexamethasone 0.1% (Alcon Laboratories Inc., Fort Worth, Texas, USA) 6 times daily for 7 days and lubricating drops as needed. One week postoperatively, loteprednol 0.5% (Bausch and Lomb Inc.) was started 5 times daily, slowly tapered over 5 weeks.

Preoperatively and at different follow-up visits, all patients received detailed ophthalmic evaluation, including UDVA, CDVA, manifest and cycloplegic refraction, slit-lamp and fundus examination, IOP measurement, full-map pachymetry, and topography (Wavelight Allegro Oculyzer).

The statistical analysis was performed using SPSS software. The Wilcoxon rank test was used to compare the dataset pre- and postoperatively.

## 3. Results

**Figure 1:**
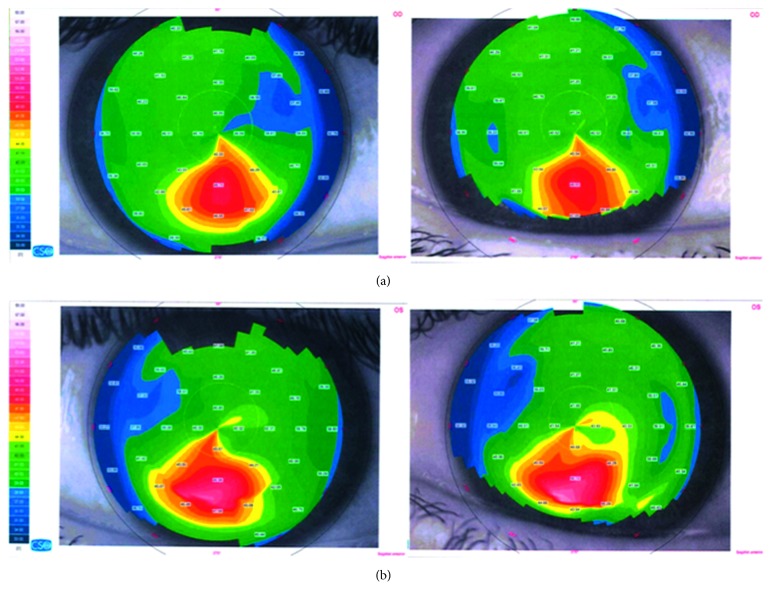
Pre- and postoperative topography of the right eye (a) and the left eye (b) of patient 1.

**Figure 2:**
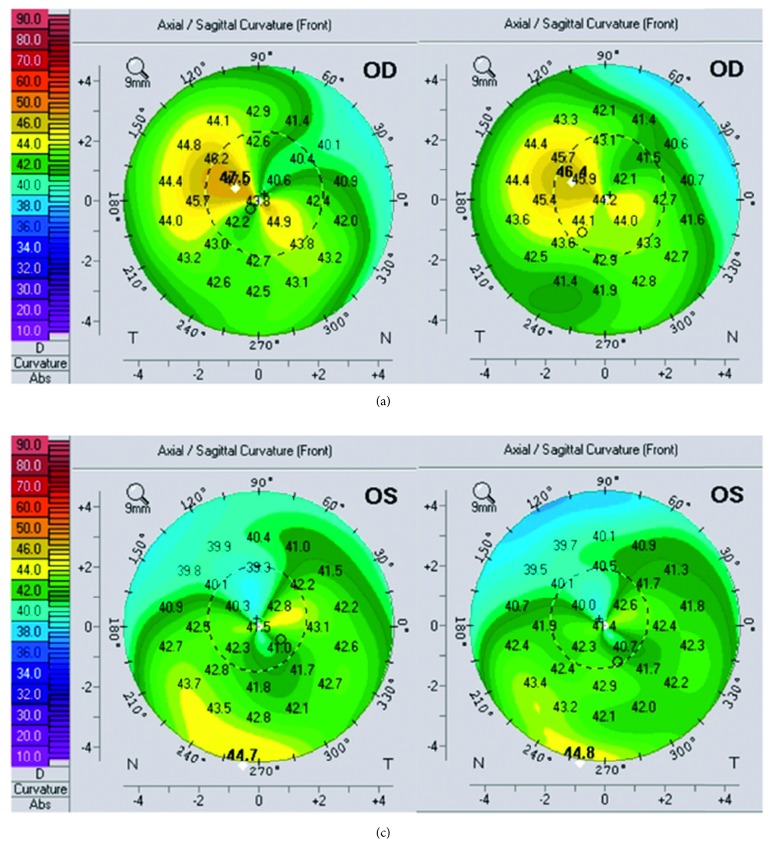
Pre- and postoperative topography of the right eye (a) and the left eye (b) of patient 2.

**Figure 3:**
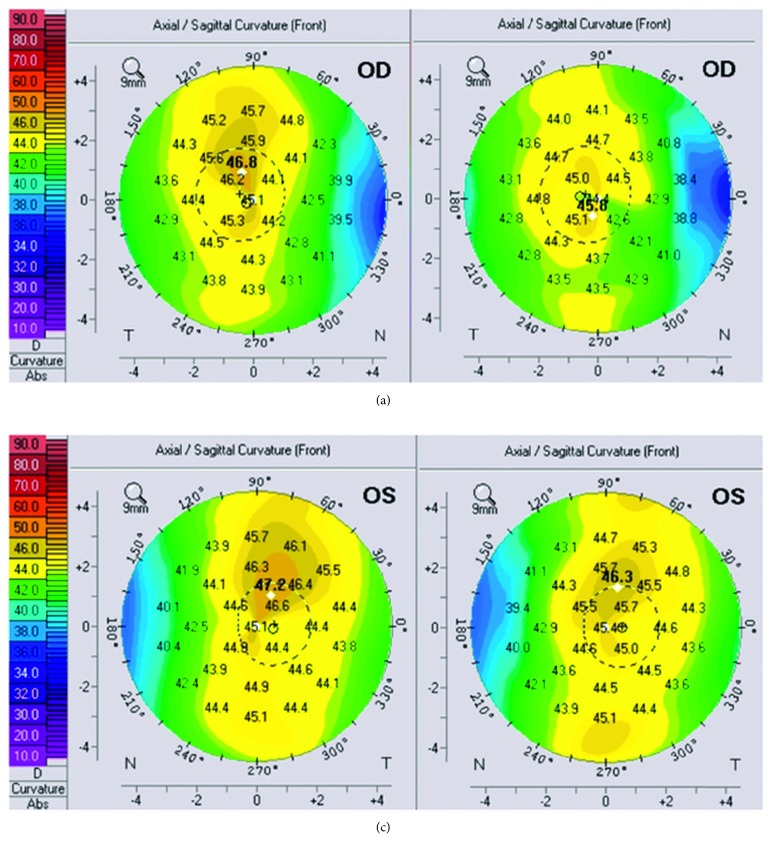
Pre- and postoperative topography of the right eye (a) and the left eye (b) of patient 3.

**Figure 4:**
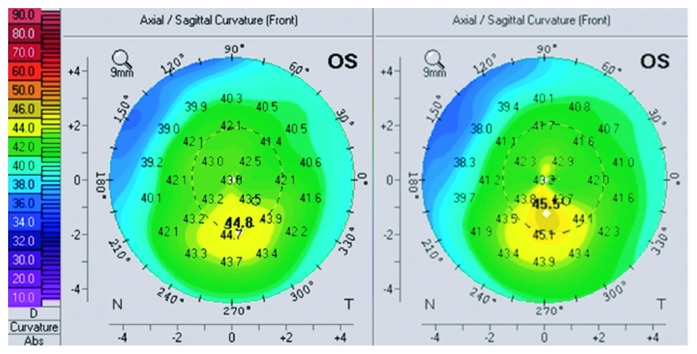
Pre- and postoperative topography of the left eye of patient 4.

Seven eyes of 4 consecutive patients (median age 21.5 years: all male) were included in the study. All patients had early ectasia with a hyperopic component of refractive error. In 4 eyes, ectasia was due to a LASIK surgery in the history and in 3 eyes due to keratoconus. All keratoconus eyes had a stage I keratoconus. Pre- and postoperative topographies for all patients are shown in Figures 1–4. Follow-up period ranged from 6 to 15 months (median 12 months). Average preoperative central corneal thickness was 547 *µ*m, which decreased to 536 *µ*m after surgery (difference of 11 *µ*m), corresponding to the average central ablation, which was 11.8 *μ*m. Mean peripheral corneal ablation was 35.15 *μ*m.

**Figure 5:**
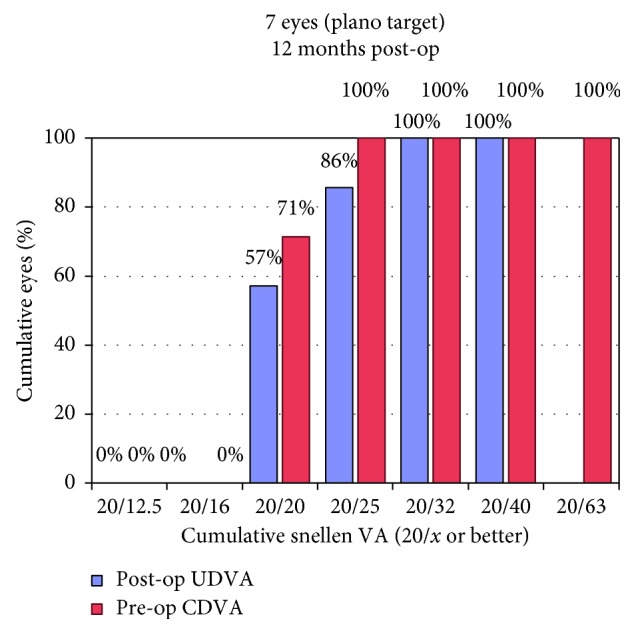
Uncorrected distance visual acuity.

**Figure 6:**
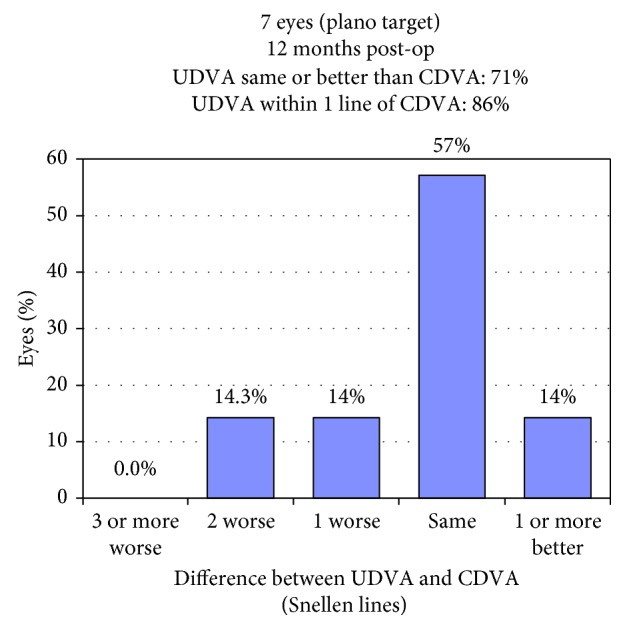
Uncorrected distance visual acuity versus corrected distance visual acuity.

**Figure 7:**
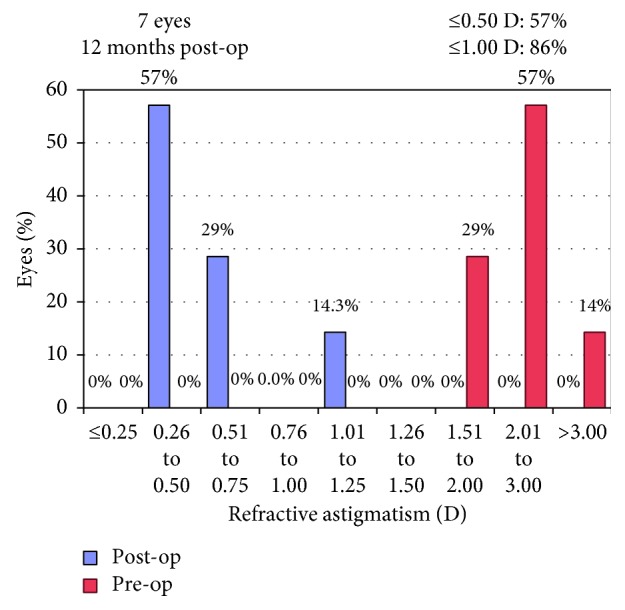
Change in corrected distance visual acuity.

**Figure 8:**
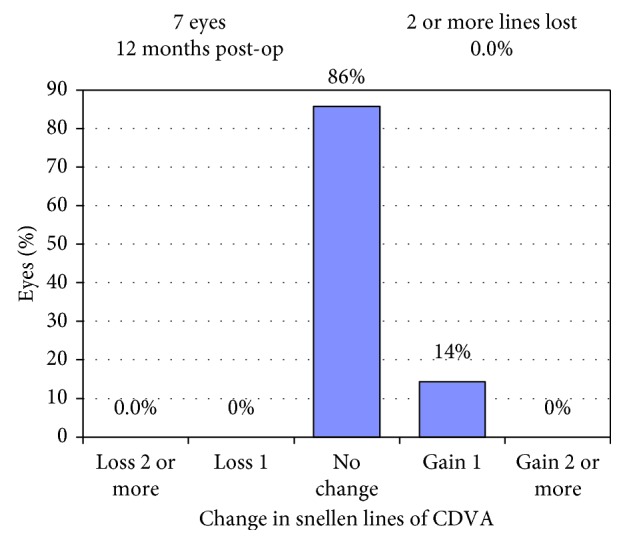
Refractive astigmatism.

Mean UDVA improved significantly from 0.35 ± 0.18 logMAR preoperatively to 0.08 ± 0.12 (*p*=0.017) at the latest follow-up. Figures 5 and 6 show postoperative UDVA in comparison to preoperative CDVA. Further significant changes were noted for cylinder, which decreased from 2.46 ± 0.53D to 0.68 ± 0.28 (*p*=0.018) (Figure 7), for SE (0.49 ± 0.60D to −0.12 ± 0.46, *p*=0.041) and for Kflat (41.3 ± 1.9D to 42.3 ± 1.4, *p*=0.028). Other parameters did not change significantly and included sphere (from −0.81 ± 0.67 to −0.46 ± 0.57, *p*=0.78), Kmax (from 47.1 ± 1.97D to 46.9 ± 2.03, *p*=0.67), and Ksteep (from 44.6 ± 1.12 to 44.3 ± 0.91, *p*=0.125). Mean CDVA preoperative was very good, 0.05 logMAR, and improved slightly to reach 0.01 logMAR (*p*=0.32) (see Figure 8). No deterioration of CDVA or UDVA was noted in any eye. No complications occurred during surgery; however, in the follow-up, two eyes of one patient developed epithelial growth within the interface, in one eye being clinically significant. For this eye, ingrowth was successfully removed. The safety index (mean postoperative CDVA (decimal)/mean preoperative CDVA (decimal)) was 1.05, as CDVA did not effectively change and the efficacy index (mean postoperative UDVA (decimal)/mean preoperative CDVA (decimal)) was 0.96.

UDVA and CDVA, manifest refraction, keratometric readings, and central corneal thickness (CCT) for all eyes before and at the latest follow-up after procedure, as well as their means, are shown in Table 1. Furthermore, the table shows depth and location of laser ablation, months of follow-up, age and sex of patients, and nature of pathology. Due to different follow-up times and different causes of disease (keratoconus vs. LASIK), Table 1 also presents individual data.

## 4. Discussion

The present paper introduces a combined procedure of a corneal flap, an excimer laser ablation and an under-the-flap CXL in the same session for the treatment of early ectasia with hyperopic refractive error. Patients treated had either a hyperopic astigmatism or a mixed astigmatism with a distinctive hyperopic component. The aim was to reach a spectacle-free emmetropia and a stable cornea.

The present procedure is similar to the LASIK Xtra technique. The energy that was used, however, was higher, equivalent to the standard CXL protocol, since corneas were already ectatic.

The results obtained in our study were successful. In all eyes treated, ectasia showed no progression over the follow-up period, which ranged from 6 to 15 months (mean 11.25 months), and UDVA improved substantially, changing significantly from 0.35 ± 0.18 logMAR to 0.05 ± 0.07 logMAR (*p*=0.017), while CDVA remained relatively stable. Average manifest refraction error improved from -0.81D sphere +2.46D cylinder (+0.49D SE) to −0.46D sphere +0.68D cylinder (−0.13D SE), while only changes in cylinder and SE were significant (*p*=0.018 and *p*=0.047, respectively).

In accordance with the correction of hyperopia, we observed a significant steepening of Kflat from 41.3D to 42.3D (*p*=0.028), whereas other K-readings did not change significantly.

Concerning flap thickness and central stromal ablation in keratoconus, the literature seems to agree that the maximal stromal ablation during combined PRK and CXL in ectatic corneas has to be leveled to 50–60 *μ*m [6, 7, 21]. In order to have a better control over a flap that was as thin as 90 *μ*m, we decided to use a femtosecond laser rather than a microkeratome. Considering the thickness of the epithelium (about 50 *μ*m [22]), the corneal flap includes about 40 *μ*m of stroma, leaving us with a residual 20 *µ*m to correct for a possible additional myopic component. The same ablation limit was considered for post-LASIK ectasia, even though residual stromal bed thickness was not measured. In only one eye we exceeded this limit by 5 *μ*m and ablated 25 *μ*m, since the myopic component was relatively large (−1.75D) and corneal thickness was relatively good (516 *μ*m). In average, central ablation was 11.8 *μ*m.

The rationale behind performing a flap instead of PRK is that PRK surgery for hyperopia has been shown to have a hyperopic regression in the long term [9, 10]. A potential remodeling effect of corneal stroma after corneal debridement, as performed in PRK, can be suggested to play a role [10]. Furthermore, when compared to LASIK, PRK has a slower visual recovery, slower corneal stabilization rate, and is associated with more haze and with more discomfort [13]. As such, the flap creation or relifting in the presented procedure is expected to lead to an earlier and higher stability, less associated complications, and better predictable corrected refraction. Indeed, corrected refraction has not shown any recurrence during the follow-up in any of the treated eyes.

A significant advantage of the procedure is that the flap allows performing CXL while keeping the epithelium of the cornea intact. From a patient's perspective, keeping the epithelium is associated with less pain and discomfort and better visual function during recovery period compared to epithelium debridement before CXL. Further, corneal sensitivity was shown to recover much faster (7 days compared to 3 months) when the epithelium was not removed [23]. This is especially of concern since corneal sensitivity correlates with blinking frequency [24] and hence with lubrication of the eye, aiding in an uncomplicated recovery. On the other hand, corneal debridement can be a cause of multiple complications, such as corneal infiltrates, subepithelial haze, ulcers, and scarring [25, 26].

The irrigation time with riboflavin was chosen to be 10 minutes, reduced in comparison to the Dresden protocol (30 minutes [27]), since irrigation happened beneath the flap and increased in comparison to the IntraLase-pocket CXL (2 minutes [28]), since riboflavin was washed out subsequently. Identifying the required time of riboflavin irrigation for an effective corneal stabilization in our treatment, however, stays a pivotal question. In addition to a longer follow-up, we suggest to determine the stromal demarcation line one month after treatment. The demarcation line possibly indicates the separation between treated and untreated cornea [29]. It appears as early as 2 weeks following CXL at an approximate depth of 300 micrometers and can be visualized at the slit-lamp and with anterior segment OCT [29, 30].

Despite crosslinking and keeping the flap very thin, the risk of a keratoconus progression due to a corneal weakening after flap creation and stromal ablation has to be taken into consideration. Our results did not reveal this risk, but nevertheless patients have to be counseled about it and be committed to a regular follow-up with topography. It is planned to reassess complications and outcome at 24 months following procedure.

Last, but not least, in case of post-LASIK ectasia, the use of an existing flap is an easy and intuitive approach, broadening the applicability of this procedure.

A possible complication of this method seems to be the formation of epithelial growth into the flap interface, as it was the case in both eyes of patient 4. It has been reported, with highly varying incidence (0–20% [31]), as a complication of LASIK surgery with an increasing risk when correcting hyperopia [32]. The interface manipulation while irrigating with riboflavin and the increased time of an open flap in our surgical procedure may be suggested as possible risk factors for the postoperative growth of epithelial cells into the flap [32]. To minimize epithelial growth, we have, subsequent to the interface-application of riboflavin, irrigated the interface with saline solution.

## 5. Conclusion

The treatment of keratectasia with hyperopia and hyperopic or mixed astigmatism constitutes a challenge to the refractive surgeon. Conventional approach involves corneal stabilization with CXL followed by adjustment of spectacles. In this study, we present a new treatment modality that aims to stabilize and correct refractive error in the same session, while combining the advantages of a corneal flap and of keeping corneal epithelium intact.

Postoperative corneal stability and significant improvement in UDVA were observed in our patients and indicate a careful appreciation of satisfactory early results. A longer follow-up and larger number of patients are needed to quantify outcome success and to evaluate long-term corneal response and the actual risk of epithelium ingrowth and recurrence of ectasia.

## Figures and Tables

**Table 1:** Pre- and postoperative parameters for all eyes separately and averaged.

Preoperative								Postoperative					
Patient (age), pathology	Eye	UDVA (logMAR)	CDVA (IogMAR)	Manifest refraction (SE) (0)	K-readings (flat/steep/max) (D)	CCT (*μ*m)	Laser ablation (c/max) (*μ*m)	Follow-up (months)	UDVA	CDVA	Manifest refraction (SE)	K-readings (flat/steep/max)	CCT
1. (34), post-LAS1K	OD	0.2	0.1	0 + 2.5 × 125°(+1.25)	39.3/43.9/48.7	466	0/34.40	6	0	0	0 + 0.5 × 90°(+0.25)	40.6/44.3/48.6	476
	OS	0.3	0.1	−0.25 + 3 × 45°(+1.25)	40.5/44.4/50.0	638	3.35/41.07		0.1	0.1	−0.25 + 0.5 × 60°(+0)	42.0/44.3/50.7	607
2. (23), KC	OD	0.7	0	−1.75 + 3.25 × 140°(−0.10)	41.3/45.7/47.5	516	25.18/47.17	13	0.18	0	−1.5 + 1.25 × 150°(−0.88)	42.6/44.9/46.4	501
	OS	0.5	0	−1.35 + 2.25 × 10°(−0.25)	39.8/43.4/44.7	527	19.25/32.19		0	0	−0.25 + 0.50 × 30°(0)	40.6/42.6/44.8	519
3. (19), post-LASIK	OD	0.18	0	−125 + 2 × 105°(0.25)	43.9/45.6/46.8	586	18.7/29.28	15	0	0	−025 + 0.50 × 30°(0)	43.7/44.8/45.8	565
	OS	0.30	0	0.025 +1.75 × 75°(+0.63)	43.8/46/47.2	581	3.43/24.26		0	0	−025 + 0.50 × 80°(0)	44.4/45.4/46.3	581
4. (21), KC	OS	0.30	0	−085 + 2.5 × 55°(+0.4)	40.4/414/44.6	515	12.64/37.71	11	0.1	0	−1.0 + 0.75 × 100°(−0.63)	42.4/44.3/46.8	493
Average		0.35±0.18	0.03 ±0.05	−0.81 + 2.46 (+0.49)	41.3/44.6/47.1	547 ± 57	11.8/35.2	11.25	0.05 ± 0.07	0.01 ± 0.04	−0.46 + 0.68(−0.13)	42.3/44.4/47.1	535 ± 48
									*p*=0.017	*p*=0.32	*p*_s_=0.078, *p*_c_=0.018, *p*_SE_=0.041	*p*_kf_=0.028, *p*_ks_=0.395, *p*_km_=0.672	*p*=0.07

CCT: central corneal thickness; CDVA: corrected distance visual acuity; c: central; D: diopters; KC: keratoconus; LASIK: laser-assisted in situ keratomileus; m: male; max: maximum; *μ*m: micrometers; OD: oculus dexter; OS: oculus sinister; *p*_s_: *p*-value for sphere; *p*_c_: *p*-value for cylinder; *p*_SE_: *p*-value for spherical equivalence; SE: spherical equivalence; UDVA: uncorrected distance visual acuity.

## Data Availability

The data used to support the findings of this study are available from the corresponding author upon request.
