# Corrigendum to “Under-the-Flap Crosslinking and LASIK in Early Ectasia with Hyperopic Refractive Error”

**DOI:** 10.1155/2019/8468507

**Published:** 2019-07-14

**Authors:** Sylvain el-Khoury, Youssef Abdelmassih, Mazen Amro, Elias Chelala, Elias Jarade

**Affiliations:** ^1^Beirut Eye Specialist Hospital, P.O. Box 116-5311, Al-Mathaf Square, Beirut, Lebanon; ^2^Saint-Joseph University, Faculty of Medicine, Beirut, Lebanon; ^3^School of Medical Sciences, Lebanese University, Beirut, Lebanon; ^4^Mediclinic Dubai Mall, Dubai, UAE

In the article titled “Under-the-Flap Crosslinking and LASIK in Early Ectasia with Hyperopic Refractive Error” [1], there was a missing reference [2] that should have been included in the reference list as reference [33]. Accordingly, the fifth paragraph in Introduction should be updated as follows:

“To prevent the development of post-LASIK ectasia in the eyes at high risk, Kanellopoulos et al. introduced the LASIK Xtra technique (Avedro, Massachusetts, USA) in 2012 [14]. In this technique, prophylactic high irradiation CXL is performed in adjunction to the LASIK procedure and without deepithelisation. Several studies demonstrated this procedure to be safe and effective, to show less regression and to have less discomfort [15–17]. A recent study has described a similar technique for the eyes that have already developed a post-LASIK ectasia. The old LASIK flap is lifted again, and the CXL is performed under the flap. This procedure keeps the epithelium intact, and preliminary results are promising [33].”

This error was brought to the authors' attention in response to a letter received on their article [3].
